# Medical News

**Published:** 1852-07

**Authors:** 


### MEDICAL NEWS.

*Extract from an Address delivered by* Dr. SAMUEL GRIMES, of Delphi, *at New Albany, May 20th*, 1852, *before the Indiana Medical Convention.*

"Before we blame with asperity the wrong bias of a portion of the community towards medical heresies and quackery, we, of the profession, ought to have the consciousness that we are clearly distinguishable from the whole tribe of pretenders, by our superior attainments, not only in medicine, but in general literature and science, as well as by our greater readiness of resource at a time of difficulty and danger in the sick room. The mere fact of our holding a diploma in Latin, while others put up with one in English, and of our believing in one doctrine, while they are clamorous in favor of another, will give us no valid claim on the world for higher professional rank, and greater skill in *practice.* We must rest our claims on something more substantive and appreciable than this. We may dwell with allowable pride on the antiquity, and learning, and signal philanthropy of the great family, with Hippocrates at its head, to which we *belong.* But the pride of ancestry becomes ridiculous if it is not sustained by contemporary merit. To be received in the great brotherhood and decorated with the order of merit, we ought to be able to show that we underwent early preparation by a good academic or collegiate education; and early training afterwards, by elementary medical instruction, in the office of an experienced physician, before we were enrolled and became regular and faithful attendants in a Medical School of repute and competent organization.

"Some, perhaps many of us, will confess, with regret, that we have not been able to comply with these requisites, in all their particulars. But, surely, this does not imply that we should withhold our advice and assistance, in order to remove the obstacles from the path of those coming after us, which embarrassed and impeded our own course. The times are more propitious to the zealous student now than they were in our early days; and, consequently, there is less excuse now than then for neglect, on the part of a parent, to procure for his son a good preparatory education, and on that of a physician to impart to his pupil suitable elementary instruction in medicine. Nor need any of us stand excused from the discharge of his duty to his pupil, if he received any such in his office, by the plea of his own defective attainments. However limited may be our reading and knowledge of medical literature, we can still point out to the young student a good book on each of the different branches of medicine; and early familiarize him with the qualities of drugs, and, to a certain extent, their pharmaceutical combinations—while we give him opportunities of ascertaining their therapeutical value. Though ourselves not learned in Climatology, we can, without difficulty put him in the way of observing and recording the characters of the successive seasons, and the modifications caused by the particular locality in which we reside. Though ignorant of Greek, thanks to the Sydenham Society, we can put into his hands a good translation of the admirable essay of Hippocrates on *Air*, *Water*, and *Places*, and read with our student the interesting and instructive volume on the climate of our own great valley, by Dr. *Drake*.

"So in Botany, even if we are not able ourselves to teach, we might, without much difficulty, set an example to our students of learning its elements, and exhort them to prosecute their inquiries in this direction, combining with them the study of vegetable physiology. Continually surrounded by the productions of nature, the country student has stronger incitements, and at the same time, greater facilities, to become acquainted with Natural History than the resident of a city.

"In pursuing the course here recommended, we shall discharge not only our duty to our students, but, also, to the several Medical Colleges to which they will resort after leaving us, for on the proper elementary instruction which these young men have received at our hands, will mainly depend their successful indoctrination in the higher principles and rational practice of Medicine by the professors in the schools. These gentlemen are continually embarrassed in their teachings by not knowing what foundation has been laid by the private teachers, or by those who ought to have been such; nor how far they may take for granted an acquaintance with elementary truths on the part of their youthful auditors. One professor, for fear of going beyond the comprehension of his class, contents himself with uttering the merest *common places;* another aims to bring up his hearers to his own elevated standard. If it be said that the first is the most popular lecturer, does not the fact show the unpreparedness of the students for a course of instruction such as they ought to receive, and such as an able and conscientious professor ought to be able to give.

"The dignity, learning, elevated standing, and successful teachings of the Faculties of our Medical Colleges cannot be matters of indifference to us. They are, in one sense, our representative, in another our auxiliaries; and whatever effects their reputation must re-act on the entire body of the profession. We cannot, therefore, be supposed to look with indifference on the organization of these colleges, nor be expected to give any countenance to those with the entire competency of whose Faculties we are not fully satisfied. A new Medical School cannot, like a new store, or a new manufactory, or a new line of stages, be patronized, merely because it is new, and to encourage trade and reduce prices, by competition. Nor can personal regards nor sectional pride furnish an apology for our sending our students, or otherwise giving our countenance to a school, formed in haste out of incongruous materials, and as if in a spirit of mere business speculation. The medical profession, at large, must divide the responsibility with the Medical Schools for the attainment and fitness of the graduates sent out annually from these latter, as candidates for practic and public patronage. If we send to the Medical Colleges crude materials, it cannot be expected that they should be at once fashioned into shape and harmonious proportions. If we send to them ignorant minds, we have no right to suppose that these will be instructed and taught the principles of medical logic, and philosophy in the course of a few months.

"I repeat, that we have a large share of responsibility for the manner in which Medical education is conducted. We send our students to be educated, we influence them in their selection of the school to which they go. We, therefore, mistake greatly our position, if we think that we can assume the office of judges and critics of Medical Schools without our participating in their cares and difficulties, and giving our counsel for their protection and guidance, when they come up to what we believe to be a good standard of teaching. The best assistance we can give them, is to send them students properly prepared to avail themselves of the higher course of instruction which the professors are ready to go through with. The best counsel we can offer to the professors, is for them to abide by their own convictions of duty in keeping up an elevated standard of medical attainments for their students, and taking adequate time for imparting their knowledge to these latter. Not only are there more branches of medicine to be taught now, but each branch is much richer in details, than formerly; and hence it is clear that more time is required to teach them than formerly was requisite.

"It may be asked, whether we are ourselves, either by education or subsequent reading, competent judges, in the premises, of what is necessary on the score of Medical Education? It may be that many of us are not, in this sense *competent*, but the very consciousness of deficiences, and experience of what we have suffered in consequence may naturally impel us to seek reform, and create a desire that others coming after us should enjoy advantages of which we were deprived.

"We may, however, all of us, in this age of periodical literature and abundant publications, readily procure the desired information on many subjects of immediate interest. The calls on our time and attention in the cares of practice, and especially country practice, must always prevent our being regular and systematic readers: but if we are bent on turning all the odd hours to account, and have a good medical journal, and the best monograph at our elbow, we shall be able to keep up, to a respectable extent, with the progress of our science, at least so far as to appreciate its extent and chief bearings, and the improvements in practice. In this way we can do justice to ourselves and to our students, and confer, if need be, understandingly with our brethren, the members of the Faculty of Medical Colleges.

"In order to give effect to our wishes and intentions on all that relates to the improvement and elevation of our profession, we must act through our State Society *organization.* Indiana, in this respect, will not, I hope, be backward in rivaling her sister States—some of which, as Pennsylvania, New York and Tennessee, furnish, in their transactions, commendable examples for imitation. For this purpose it is all important that the County Societies should be well organized—as on their efficiency will depend the weight and character of the State Society. From each of them we may expect an account of the medical topography of the county, and of the diseases to which it is subject in successive seasons, together with those of an evidently endemic nature. Clearness and brevity should be consulted in these descriptions and histories, if it is desired to render them acceptable to hearers or readers, and creditable to their authors.

"I would recommend our Society to petition the State Legislature for an act requiring a registration of births, deaths, and marriages, so that we may be furnished with valuable *vital statistics*, and which will serve as a basis for many exceedingly interesting inquiries both of medical and general value.

"As far as lies in our power we shall, it is to be hoped, be, all of us, willing to give weight and efficiency to the recommendations of the National Medical Association, to which we are already indebted for an excellent Code of Ethics, and for various Reports on the Literature and Practice of Medicine, and the means of elevating the standard of Medical Education. If the State and County Societies act in unison with the National Association or *Congress*, the best results will follow, not only to our profession, but to the nation at large."

Present to the Royal College of Surgeons.—Mr. Abbott Lawrence, the United States Minister at London, having stated to the Lords Commissioners of her Majesty's Treasury that two cases have arrived from New York, addressed to him, and containing fossils sent by Dr. John C. Warren of Boston, United States, for presentation to the Royal College of Surgeons, London,—their Lordships have given directions to the proper authorities of the revenue to permit their free delivery for the purpose stated.—*London Lancet.*

Opposition to Vaccination.—Such is either the unwillingness or carelessness of parents regarding the vaccination of their children, in Great Britain, that medical men are compelled to seek the interference of clergymen to induce parents to have their children vaccinated. The Irish especially are opposed to the operation.—*Ibid.*

The Concours for the Chair of Hygiene, in the Faculty of Medicine of Paris, which we noticed in our Journal of March 6, has terminated with the appointment of M. Bouchardat, Pharmacien-en-Chef to the Hôtel Dieu, to the vacant professorship. We learn, also, that M. le Docteur Dupré has recently been elected to the Chair of Medicine in the Faculty of Medicine of Montpellier.—*Lond. Med. Times.*

Progress of Epidemics.—The small-pox is spreading in Jamaica. In the parish of St. Anne, where it had only been prevalent two or three weeks, there were upwards of 4000 cases. The greatest destitution prevailed, and, in many instances, poor persons perished for the want of assistance and of medical care. In Kingston there are several cases. The cholera, which was lately reported to be exceedingly fatal at Bokhara, is now stated to have broken out in some places in Upper Silesia, a part of the Prussian kingdom, where great misery prevails. If this be really the case, and it should extend, as it will in all probability, the mortality will be dreadful, for the dearth and misery in many parts of Germany have, of late, been on the most fearful scale, so that the bodies of dead dogs, in a semi-putrescent state, have been devoured by the starving inhabitants.—*Ibid.*

OBITUARY.—Dr. JOHN DALRYMPLE, the able microscopist and oculist, of London, died on the 2nd of May last.

A medical man in Breslau has been fined 40 thalers for having neglected to attend to a patient who sent for him. (!)

M. CHOMEL.—Politics even affect the peaceable science of Medicine. M. Chomel, the celebrated Parisian physician, having refused to take the oath to the President, required by the *quasi*-Constitution, has vacated his Professorship at the College of Medicine.

